# Reuse of unused medications: a cross-sectional study on public willingness and influencing factors

**DOI:** 10.3389/jpps.2025.15249

**Published:** 2025-12-01

**Authors:** Faten Alhomoud, Sakinah Alalwyat, Lama Alanzi, Farah Kais Alhomoud, Sarah Khayyat, Khalid A. Alamer, Basmah Alfageh, Mohra Aladwani, Abdullah A. Alhifany

**Affiliations:** 1 Department of Pharmacy Practice, College of Pharmacy, Imam Abdulrahman Bin Faisal University, Dammam, Saudi Arabia; 2 College of Pharmacy, Imam Abdulrahman Bin Faisal University, Dammam, Saudi Arabia; 3 Department of Pharmaceutical Practices, College of Pharmacy, Umm Al-Qura University, Makkah, Saudi Arabia; 4 Department of Clinical Pharmacy, College of Pharmacy, King Saud University, Riyadh, Saudi Arabia; 5 Department of Clinical Pharmacy, College of Pharmacy, Taif University, Taif, Saudi Arabia

**Keywords:** medicine waste, medicine re-dispensing, medicine reuse, unused medicine, recycle

## Abstract

Medication waste is a significant global concern with environmental, economic, and healthcare implications. In Saudi Arabia, approximately 25.8% of dispensed medications are wasted, resulting in an annual cost of $150 million. Re-dispensing unused medications has been proposed to reduce this waste; however, its feasibility depends on public acceptance, regulatory frameworks, and assurances of safety. This study aimed to assess the Saudi public’s willingness to accept re-dispensed medications returned unused to pharmacies and to identify factors influencing this willingness. A descriptive cross-sectional survey was conducted online across Saudi Arabia. The questionnaire, adapted from a validated tool by McRae et al. McRae et al. (Pharmacy (Basel), 2021, 9(2): 77) and translated into Arabic, explored demographics, medication practices, storage and disposal, and attitudes towards medication waste and re-dispensing. The survey was distributed via social media. Data were analyzed using SPSS version 29, including chi-squared tests and binary logistic regression. A total of 405 participants completed the survey, primarily female (64%) and aged 25–44 years (43%). About 64% reported having unused medications at home, most commonly stored in bedrooms (55.1%) and kitchens (53.6%). Disposal practices included keeping medicines for future use (62.5%), discarding them with household waste (45.7%), sharing them with others (21.5%), and returning unused medications to a pharmacy (8.4%). Approximately 60% were willing to accept re-dispensed tablets and 55% capsules, whereas fewer accepted other dosage forms. Key factors influencing acceptance included pharmacist verification of quality and integrity (79.3%), informed consent (77.3%), expiry dates (77%), and intact packaging (74.8%). Most participants (68.1%) indicated they would return unused medicines if a re-dispensing program were implemented, and half (50.6%) believed all medications, not only expensive ones, should be considered. Significant predictors of willingness included age (P < 0.001), employment status (P = 0.004), regular prescription use (P = 0.046), and concern about waste (P < 0.001). Younger participants showed higher acceptance, while employed individuals, retirees, and regular medication users were more hesitant. The findings indicate cautious yet notable public support for medication re-dispensing in Saudi Arabia, particularly for oral solid dosage forms, provided rigorous safety measures are assured. Policymakers should consider these insights to guide initiatives aimed at reducing medication waste.

## Introduction

Up to one-third of patients do not consume all the medicines dispensed by their pharmacy [1]. Patients either return unused medicines to pharmacies, where they are treated as special waste and discarded, or dispose of them at home as household waste [2]. This may result in medicinal waste, loss of healthcare resources, and environmental pollution in cases of incorrect medication disposal [3–5]. The annual economic impact of medication waste among Saudi families is estimated to be $150 million annually [6]. Medication waste in Saudi Arabia accounts for 25.8%, compared to 41.3% in other Gulf countries [6]. A substantial proportion of medicines [ranging from 20% to 90%] are returned to the pharmacy in their original, unopened packaging [7–11]. Thus, some healthcare systems have proposed the re-dispensing of returned and unused medication [7–14].

Re-dispensing medications involves reissuing medications that have been returned unused by patients or their families, typically within healthcare settings such as hospitals, pharmacies, or nursing homes [7–14]. However, the success of such initiatives depends on the following [12, 13]: 1) the willingness of patients to return unused medication to the pharmacy and accept re-dispensing of unused medicines for their treatment; 2) the availability of legal authorization and clear guidelines on which medicines can be re-dispensed, and the circumstances under which this can occur; 3) the adaptation of verification procedures, quality assurance processes, or advanced packaging technologies that could notify pharmacists if medicines have been improperly stored, handled, or tampered with, so the medication is in good condition and meets all safety criteria; and 4) the inclusion of visible expiry dates on the drug’s packaging to verify its validity and safety.

Although reusing unused returned medicines could lead to waste reduction and environmental protection, this concept remains widely debated [7–13]. Concerns about safety, product quality, effectiveness, and psychological discomfort associated with the use of medications previously owned by others are the reasons why re-dispensing is not widely adopted as a standard practice in most countries [13]. The Saudi Ministry of Health currently considers the reissue of medicines unethical and prohibits this practice. Similarly, the World Health Organization’s guidelines for drug donations also discourage sending unused medicines from one country to another unless medications meet specific criteria, such as being in their original container, unopened packaging, and having a long shelf life. These guidelines emphasize the importance of ensuring that donated medications are both effective and safe [14]. Despite this, the possibility of reusing medications has been discussed several times. A charity in the United Kingdom (UK) has also been re-dispensing patients’ unused medicines for humanitarian aid [15]. In the United States (USA), for instance, unused medications are collected and redistributed to patients who cannot afford the cost of medication [16]. In Italy, a return and reuse medication program was implemented for 3 years, demonstrating the benefits of reusing medication, primarily in reducing costs [17]. Studies in the UK and the Netherlands found that people would accept the re-dispensing of unused medicines if product quality and safety were guaranteed [13, 18–20]. In line with these international experiences, recent evidence from Jordan found that nearly three-quarters of the public expressed willingness to reuse unused medicines if safety and quality were guaranteed, with economic and environmental benefits cited as key motivators [21]. Beyond public perspectives, healthcare providers and stakeholders have highlighted the importance of tamper-evident packaging, digital monitoring of storage conditions, and clear legal frameworks to ensure feasibility [18, 22–24]. These insights suggest that while medication reuse has the potential to reduce waste and support sustainability, its success depends on robust safety verification, professional oversight, and alignment with international and national regulations. This could potentially address both preventable and non-preventable causes of medication waste. Preventable causes include patient stockpiling, while non-preventable causes include patient death, recovery, or a change in treatment [24].

Medication waste imposes considerable economic, environmental, and healthcare burdens [2–6]. While international initiatives in countries such as the UK, USA, Italy, and the Netherlands have explored medication reuse, and regional evidence from Jordan has demonstrated public willingness under strict safety conditions, no study has yet examined this issue in Saudi Arabia. Understanding public attitudes and the factors influencing acceptance of re-dispensed medications is essential for informing national policy, reducing waste, and ensuring patient safety. This study, therefore, fills a critical gap by providing evidence specific to the Saudi context, with implications for sustainable healthcare practices and regulatory development. The aim of this study is to assess the public’s willingness to use unused medicines returned to the pharmacy by other patients and the characteristics associated with this willingness. Thoroughly understanding patients’ willingness to use returned, unused medications is essential for assessing the feasibility of re-dispensing them.

## Methods

### Study design

A descriptive cross-sectional survey was conducted in Saudi Arabia (KSA) over a 2-month period, from December 2024 to January 2025.

### Data collection and sample

Data were collected anonymously using the QuestionPro platform and securely stored on password-protected systems, with access restricted to the research team only. Participants were required to provide informed consent before commencing the survey. This study used a convenience sampling technique. All individuals aged 18 years and older residing in the KSA who speak either Arabic or English were considered eligible for inclusion in the study. Individuals without access to the Internet, social media, or illiterate people were excluded from the study. The survey was distributed through social media (e.g., Facebook, Twitter, WhatsApp, Telegram, and LinkedIn).

### Sample size

The sample size was calculated using the Raosoft sample calculator. Considering that the population in Saudi Arabia aged 18 and above is approximately 25 million [25], the sample size was calculated using a 5% margin of error, 95% confidence level, and 50% response distribution. This resulted in a final sample size of 385. However, we ultimately received 405 complete responses. Since all responses met the inclusion criteria, we included the entire dataset in the analysis. This approach enhanced the statistical power and robustness of the findings.

### Study questionnaire and its translation

The questionnaire used in this study was adapted from a survey developed by McRae et al. [26], which assessed public attitudes towards medicinal waste and the reuse of prescription medicines. Slight modifications were made to suit the context of this study, particularly for the personal information section, where the demographic variables were expanded to include region of residence, monthly income in Saudi Riyals, presence of healthcare providers in the family, and household size. In addition, the original 5-point Likert scale (ranging from strongly agree to strongly disagree) was simplified to a 3-point scale (agree, neither, disagree) with an additional don’t know option, in order to improve clarity and ease of response. Minor wording adjustments were also made to ensure cultural and linguistic appropriateness. These modifications preserved the validity of the original tool while tailoring it to the Saudi context. The adapted questionnaire retained key elements that explore medication use, beliefs about medicinal waste, storage, disposal practices, and acceptability of re-dispensing returned medications.

The questionnaire consisted of five sections [1]: personal information [2], medication use information [3], beliefs about medication waste [4], storage and disposal of medication, and [5] beliefs about re-dispensing unused prescription medications. Each section included closed-ended questions, such as multiple-choice, yes/no, Likert-scale, and open-ended questions. Respondents were required to answer all questions, except for the open-ended questions.

The original English version of the questionnaire was translated into Arabic using a parallel translation method in which two independent translators worked on the translation separately. The translations were compared. Any discrepancies were resolved by discussion, and a final version was created. This was followed by a face validity assessment conducted by experts to ensure the clarity and relevance of the questions. Pre-testing was also conducted with a small group of participants to identify potential issues and refine the questionnaire prior to its full implementation.

### Data analysis

Descriptive statistics were used to summarize participants' characteristics and responses to the questionnaire items. Frequencies and percentages were reported for categorical variables, whereas means and standard deviations were reported for continuous variables, where appropriate.

The Chi-square test was conducted to assess the associations between willingness to reuse unused prescription medications and various participant characteristics (such as age, gender, education level, and employment status). Logistic regression analysis further explored the relationship between demographic factors and willingness to reuse unused medications. The results are presented as odds ratios (ORs) with 95% confidence intervals (CIs). The logistic regression model helped to adjust for potential confounders and determine which factors were independently associated with willingness to reuse medications. All statistical analyses were performed using SPSS (Statistical Package for the Social Sciences) version 29, and a P-value of less than 0.05 was considered statistically significant.

### Ethical approval

This study was conducted in accordance with the ethical principles outlined in the Declaration of Helsinki. Ethical approval from the Institutional Review Board (IRB) committee of Imam Abdulrahman Bin Faisal University was obtained before this study commenced (IRB-2024-05-762). At the start of the survey, all participants provided written informed consent, covering voluntary participation, data confidentiality, and permission for publication.

## Results

### Study response and participants’ characteristics

The study included 405 participants, of whom 64% were female and 43% were aged between 25 and 44 years. The majority were Saudi nationals (96%) and held bachelor’s degrees (54.6%). In terms of employment, 41.7% of respondents were employed. Most respondents were from non-medical professions (77.5%) and resided in the Eastern Province (79%). Private health insurance was held by 55.6% of the participants, and 60% reported having a family member in the healthcare field. Household size data showed that 47.2% lived with four to six members, and 38.1% lived with more than 6. Table 1 provides a detailed description of participants’ characteristics.

**TABLE 1:** Characteristics of participants recruited into the study (Total Number = 405).

Variable	N	%
Gender		
Male	146	36
Female	259	64
Age		
18–24	13	33.8
25–44	174	43
45 and above	94	23.2
Nationality		
Saudi	389	96
Non-Saudi	16	4
Level of education		
High school or below	92	22.7
Diploma’s degree	57	14.1
Bachelor’s degree	221	54.6
Postgraduate studies (Master’s or Ph.D)	35	8.6
Employment status		
Employed	169	41.7
Self-employed	17	4.2
Unemployed	72	17.8
Retired	29	7.2
Student	118	29.1
Profession		
Medical profession	91	22.5
Non-medical profession	314	77.5
Income per month		
<8000 SAR	226	55.8
8,000-16000 SAR	113	27.9
>16,000 SAR	66	16.3
Private health insurance		
Yes	255	55.6
No	180	44.4
Province		
Eastern province	320	79
Other provinces	85	21
Healthcare provider in the family		
Yes	243	60
No	162	40
Number of the people in the house		
1–3	58	14.6
4–6	187	47.2
>6	151	38.1

(N = number; % = percentage; SAR, Saudi Arabian Riyal).

### Medication use and waste

Approximately 48.9% of respondents reported regularly taking medications. Among them, 35.8% took 1–3 medications, and 8.1% took four or more medications. Unused medication at home was reported by 64% of the respondents. When asked about concerns regarding medical waste, 66.4% agreed that they were concerned, 21.7% were neutral, and only 6.7% disagreed. See Table 2.

**TABLE 2:** Medication use patterns and concern about medicine waste.

Variable	N	%
Regular prescribed medication		
Yes	198	48.9
No	207	51.1
Number of regular prescription medications		
None	227	56
1–3	145	35.8
>=4	33	8.1
Unused medication at home		
Yes	259	64
No	146	36
Concern about prescription medication waste		
Agree	269	66.4
Neutral	88	21.7
Disagree	27	6.7
Don’t know	21	5.2

### Storage, disposal, and perceptions of returned medications

Medicines were most commonly stored in bedrooms (55.1%) and kitchens (53.6%), while bathrooms and entrance halls were used less frequently (7% each). A small percentage (3.7%) of patients did not receive any medications. Regarding disposal, 62.5% kept unused medications for future use, 45.7% discarded them with household waste, 21.5% shared them with others, and only 8.4% returned them to a pharmacy for disposal. See Table 3.

**TABLE 3:** Storage locations, disposal practices, and perceptions of returned medications.

Variable	N	%
Medicine storage location^a^		
Living room	75	18.5
Kitchen	217	53.6
Bathroom	3	7
Bedroom	223	55.1
Entrance hall	3	7
No medicines	15	3.7
Disposal Practice^a^		
Throw out with household waste	185	45.7
Keep for future use	253	62.5
Return to pharmacy	34	8.4
Share with others	87	21.5
Don’t use medicines	24	5.9
Perceptions of returned medications		
Re-dispensed to other people	90	22.2
Sent to developing countries	34	8.4
Destroyed	99	24.4
Not sure	189	44.9

^a^ Participants were allowed to select multiple responses; therefore, percentages may exceed 100%.

When asked about what happened to return medications, 44.9% were unsure. A quarter (24.4%) believed that they were destroyed, while 22.2% thought they were re-dispensed. Only 8.4% believed that they were sent to third-world countries. See Table 3.

### Acceptance of re-dispensed medications and influencing factors

Participants showed the highest acceptance of oral solid dosage forms, such as tablets (60%) and capsules (55%), followed by skin patches (54%). In contrast, there was rejection for pessaries (93%), injections (89%), and suppositories (87%), indicating a clear preference for noninvasive forms. Other forms, such as creams, nasal sprays, eye drops, and ear drops, received modest acceptance (approximately 26–34%) among participants. See Table 4.

**TABLE 4:** Acceptance of re-dispensed dosage forms.

Dosage form	Acceptance (yes)		Rejection (no and unsure)	
	N	%	N	%
Liquid medicines	63	16	342	84
Inhalers	77	19	328	81
Tablets	243	60	162	40
Capsules	223	55	182	45
Creams or ointments	136	34	269	66
Suppositories (medicines that are inserted into the rectum)	53	13	352	87
Pessaries (medicines that are inserted into the vagina)	30	7	375	93
Injections	44	11	361	89
Skin patches	220	54	185	46
Nasal sprays or nose drops	105	26	300	74
Eye drops/eye ointments	110	27	295	73
Ear drops	118	29	287	71

Key factors considered essential for accepting re-dispensed medicines included pharmacist verification (79.3%), patient consent (77.3%), the medicines being in date (77%), intact tamper-proof seals (74.8%), and clean packaging (71.1%). Participants also emphasized the importance of receiving adequate information about the re-dispensed medication (76.8%) and strongly preferred that the products remain unopened and visibly safe. See Table 5.

**TABLE 5:** Determinants of acceptance.

Factor/Condition	Essential		Desirable		Unsure		Not needed	
	N	%	N	%	N	%	N	%
Pharmacist verification of the medication	321	79.3	37	9.1	28	6.9	19	4.7
Patient informed consent	313	77.3	43	10.6	19	4.7	30	7.4
The medication is within the expiration date	312	77	37	9.1	31	7.7	25	6.2
Being informed that the medication is re-dispensed	311	76.8	30	7.4	31	7.7	33	8.1
Tamper-proof or intact packaging	303	74.8	47	11.6	36	8.9	19	4.7
Clean and undamaged packaging	288	71.1	39	9.6	46	11.4	32	7.9
None of the tablets/capsules in the blister pack have been used	223	55.1	95	23.5	42	10.4	45	11.1

### Attitudes toward reuse and its impact

Participants expressed concern about the improper storage of returned medicines (71.9%), and 45.2% were worried about potential fraud. However, 61.2% recognized environmental benefits and 59.5% saw economic advantages. While 35.6% believed that it was safe to use returned medicines, 38.3% thought they may be ineffective. See Table 6.

**TABLE 6:** Attitudes toward medication reuse and perceived impact.

Attitude statement	Agree		Neutral		Disagree		Don’t know	
	N	%	N	%	N	%	N	%
Concerned about improper storage of returned medicines	291	71.9	51	12.6	26	6.4	37	9.1
Believe reuse is environmentally beneficial	248	61.2	86	21.2	29	7.2	42	10.4
Believe reuse saves money and reduces healthcare costs	241	59.5	99	24.4	29	7.2	36	8.9
Concerned about potential fraud	183	45.2	75	18.5	63	15.6	84	20.7
Believe it is safe to use returned medications	144	35.6	133	32.8	72	17.8	56	13.8
Believe that returned medicines may be ineffective	155	38.3	101	24.9	79	19.5	70	17.3
Medicine packs that have been returned unused should be destroyed	88	21.7	134	33.1	142	35.1	41	10.1

### Willingness to participate in reuse programs

If re-dispensing programs were implemented, 68.1% stated that they would be more likely to return unused medicines (Table 7). However, 59.5% never returned (Table 8). Half (50.6%) believed that all medicines should be eligible for re-dispensing, while 25.7% supported re-dispensing only expensive medications (Table 9).

**TABLE 7:** Willingness to return unused medicines.

Statement	N	%
Would more likely return if reuse program exists	276	68.1
Would less likely return if reuse program exists	66	16.3
Would not change how I get rid of medicines if reuse program exists	63	15.6

**TABLE 8:** Frequency of returning unused medicines back to the pharmacy.

Frequency category	N	%
Always	22	5.4
Often	24	5.9
Sometimes	45	11.1
Rarely	73	18
Never	241	59.5

**TABLE 9:** Public preferences for types of medicines to be re-dispensed.

Preference	N	%
All medications	205	50.6
Only expensive medications (perhaps costing the ministry of health greater than 100 SAR) should be considered for re-dispensing	104	25.7
Not sure	96	23.7

### Statistical associations with willingness

Chi-square tests indicated significant associations between willingness to use returned medicines and age (P < 0.001), employment status (P = 0.004), regular prescription use (P = 0.046), number of medications (P = 0.027), and concern about waste (P < 0.001). See Table 10.

**TABLE 10:** Bivariate associations between participant characteristics and willingness to use re-dispensed Medications.

Variable	Willing (agree) (%)	Not willing (disagree/Neutral/Don’t agree) (%)	X^2^	df	P-value
Gender					
Male	37.7	62.3	0.446	1	0.504
Female	34.4	65.6			
Age					
18–24	26.3	73.7	18.281	2	<0.001
25–44	33.3	66.7			
45 and above	53.2	46.8			
Level of education					
High school or below	31.5	68.5	6.901	3	0.075
Diploma’s degree	49.1	50.9			
Bachelor’s degree	32.6	67.4			
Postgraduate studies (Master’s or Ph.D)	42.9	57.1			
Employment status					
Employed	40.2	59.8	15.626	4	0.004
Self-employed	64.7	35.3			
Unemployed	31.9	68.1			
Retired	44.8	55.2			
Student	24.6	75.4			
Profession					
Medical profession	34.1	65.9	0.114	1	0.736
Non-medical profession	36	64			
Income per month					
<8000 SRA	33.2	66.8	1.286	2	0.526
8,000-16000 SRA	38.1	61.9			
>16,000 SRA	39.4	60.6			
Private health insurance					
Yes	36	64	0.044	1	0.835
No	35	65			
Province					
Eastern province	36.3	63.7	0.321	1	0.571
Other provinces	32.9	67.1			
Healthcare provider in the family					
Yes	35	65	0.088	1	0.767
No	36.4	63.6			
Number of the people in the house					
1–3	27.6	72.4	4.296	2	0.117
4–6	32.6	67.4			
>6	41.1	58.9			
Regular prescription use					
Yes	40.4	59.6	3.974	1	0.046
No	30.9	69.1			
Number of regular prescription medications					
None	30	70	7.202	2	0.027
1–3	42.1	57.9			
>=4	45.5	54.5			
Unused medication					
Yes	37.5	62.5	1.127	1	0.288
No	32.2	67.8			
I Am concerned by the amount of prescription medicines which are wasted					
Agree	41.3	58.7	11.392	1	<0.001
Neutral/Disagree/Don’t know	24.3	75.7			

(X^2^ = Chi-square statistic, df = Degrees of freedom, P-value = Probability value indicating statistical significance).

Logistic regression analysis showed that younger participants were significantly more willing to accept re-dispensed medications than those aged 45 years and above. Specifically, participants aged 18–24 years had more than twice the odds ratio (OR = 3.188, 95% CI: 1.829–5.558, P < 0.001), and those aged 25–44 years had more than twice the odds ratio (OR = 2.273, 95% CI: 1.360–3.797, P = 0.002) compared to the reference group. Compared to students, employed (OR = 0.484, 95% CI: 0.288–0.814, P = 0.006), self-employed (OR = 0.178, 95% CI: 0.060–0.523, P = 0.002), and retired individuals (OR = 0.401, 95% CI: 0.173–0.932, P = 0.034) were significantly less willing. Regular users of prescribed medications were also less likely to accept reused medicines (OR = 0.660, 95% CI: 0.438–0.994, P = 0.047). Interestingly, those who expressed concern about medication waste were also less likely to accept reuse (OR = 0.456, 95% CI: 0.288–0.723, P < 0.001). No significant associations were observed for gender, education, income, profession, location, household size, or having a healthcare provider in the family. See Table 11.

**TABLE 11:** Predictors of willingness to use re-dispensed medications (Binary logistic regression analysis).

Parameter		Binary logistic	
		OR (95% CI)	P-value
Gender	Male	0.866 (0.568–1.321)	0.504
	Female	Reference	
Age	18–24	3.188 (1.829–5.558)	<0.001
	25–44	2.273 (1.360–3.797)	0.002
	45 and above	Reference	
Level of education	High school or below	1.629 (0.731–3.630)	0.232
	Diploma’s degree	0.777 (0.333–1.812)	0.559
	Bachelor’s degree	1.552 (0.751–3.208)	0.235
	Postgraduate studies (Master’s or Ph.D)	Reference	
Employment status	Employed	0.484 (0.288–0.814)	0.006
	Self-employed	0.178 (0.060–0.523)	0.002
	Unemployed	0.694 (0.363–1.328)	0.270
	Retired	0.401 (0.173–0.932)	0.034
	Student	Reference	
Profession	Medical profession	1.088 (0.666–1.778)	0.736
	Non-medical profession	Reference	
Income per month	<8000 SRA	1.309 (0.743-2.305	0.352
	8,000-16000 SRA	1.058 (0.568–1.973)	0.859
	>16,000 SRA	Reference	
Private health insurance	Yes	0.957 (0.635–1.442)	0.835
	No	Reference	
Province	Eastern province	0.864 (0.521–1.434)	0.571
	Other provinces	Reference	
Healthcare provider in the family	Yes	1.065 (0.703–1.612)	0.767
	No	Reference	
Number of the people in the house	1–3	1.829 (0.944-3.541	0.073
	4–6	1.439 (0.922–2.247)	0.109
	>6	Reference	
Regular prescription use	Yes	0.660 (0.438–0.994)	0.047
	No	Reference	
Number of regular prescription medications	None	1.949 (0.928–4.091)	0.078
	1–3	1.148 (0.536–2.455)	0.723
	>=4	Reference	
Unused medication	Yes	0.793 (0.516–1.217)	0.289
	No	Reference	
I Am concerned by the amount of prescription medicines which are wasted	Agree	0.456 (0.288–0.723)	<0.001
	Neutral/Disagree/Don’t know	Reference	

(OR, odds ratio; CI, confidence interval, P-value = Probability value indicating statistical significance).

The final logistic regression model explained approximately 20% of the variance in willingness to use re-dispensed medications (Nagelkerke R^2^ = 0.20), indicating a moderate model fit.

## Discussion

This study examined public attitudes in Saudi Arabia toward the reuse of unused medications returned to pharmacies, revealing both cautious support and significant influencing factors. Approximately 60% of our participants expressed willingness to accept re-dispensed tablets and capsules, aligning with the international literature. Bekker et al. [13] in the Netherlands reported a similar acceptance rate (61.2%) when quality was guaranteed, while McRae et al. [26] found even higher willingness in Wales, with 78.7% and 75.1% acceptance of tablets and capsules, respectively. The preference in our study for non-invasive dosage forms such as tablets and capsules, and rejection of injections or pessaries mirrors these earlier findings [26]. Alhamad et al. [21] also observed higher public acceptance for reusing oral medications than other forms, emphasizing that dosage form greatly affects public trust in reused medications. Although the healthcare systems in the Netherlands and Wales differ from that of Saudi Arabia—particularly regarding medication dispensing and reuse regulations—the comparison was drawn to highlight international patterns of public acceptance rather than direct policy equivalence.

Our participants emphasized the importance of pharmacist verification, intact packaging, expiry date visibility, and informed consent, which were also highlighted in the McRae [26] and Bekker [13] studies as essential conditions for public approval. This reinforces the idea that the successful implementation of reuse programs depends on rigorous safety protocols and transparent communication.

The results also showed that although 59.5% of the participants had never returned unused medicines to the pharmacy, most indicated that they would do so if a reuse program existed. This matches the patterns reported in Wales [26] and the Netherlands [13], where participants expressed a greater willingness to return medications when they believed the system would use them effectively. These findings suggest that, while public support for medication reuse exists in Saudi Arabia, it highly depends on safety, transparency, and trust in the pharmacy system. With proper protocols, legal backing, and public education, there is strong potential to implement effective and widely accepted medicine reuse programs.

Interestingly, 50.6% of respondents in the current study believed that all medications, not just expensive ones, should be eligible for re-dispensing—a more progressive view than that reported in the UK [26] or Netherlands [13]. McRae et al. [26] found that some participants believed free medications were less valued, which may reduce public motivation for reuse in such systems. In contrast, our findings suggest that in Saudi Arabia, environmental and social responsibility may override cost considerations, reflecting a public concern about medication waste (66.4% expressed concern).

Another key finding was the influence of demographic factors. Younger participants were significantly more willing to use re-dispensed medications than older participants. This aligns with Alhamad et al. [21], who found that younger individuals in Jordan were more open to reuse initiatives, potentially because of more flexible attitudes and greater environmental awareness. Interestingly, Bekker et al. [13] did not find age to be a statistically significant predictor, suggesting possible cultural or system-based differences. Our study revealed that being employed or retired was associated with a lower willingness to use medications that had been previously dispensed. The lower willingness observed among retired participants may be attributed to greater safety concerns and perceived vulnerability among older adults, who typically use multiple medications and may prefer newly dispensed medicines to minimize perceived risks. Similarly, Alhamad et al. [21] found that employed individuals, particularly those with higher education or in the healthcare sector, showed more skepticism about safety issues.

In our analysis, regular users of prescription medications were more hesitant to accept re-dispensed drugs, which may reflect heightened safety concerns or higher expectations of medicine quality. This contrasts with Bekker et al. [13], who found that those regularly using medications were more willing to accept reused medications, possibly because of familiarity and experience. This discrepancy may also arise from cultural attitudes toward medication safety or differences in trust levels within the healthcare system. Alhamad et al. [21] similarly observed mixed reactions among chronic medication users; while some supported reuse due to cost and environmental concerns, others worried about effectiveness and storage integrity.

In our study, concern about medication waste was a significant predictor, but contrary to expectations, those who expressed greater concern were less likely to accept re-dispensed medications. This suggests that, while people may acknowledge the issue of medication waste, their concerns about safety, storage, or quality may outweigh their willingness to participate in reuse initiatives. This finding contrasts with those of previous studies. For instance, McRae et al. [26] reported that 89.1% of participants were concerned about medicinal waste, which was associated with a greater acceptance of reuse schemes. Similarly, Bekker et al. [13] found that participants who had experience with unused medications or had returned medicines were more open to re-dispensing. Alhamad et al. [21] also observed that environmental concerns and the desire to reduce waste are key motivators for supporting medication reuse. These conflicting results highlight the need for targeted public education that not only raises awareness about the scale of medication waste but also reassures the public about the safety and regulatory oversight of re-dispensing programs. Future policies should prioritize building public trust by involving pharmacists in quality verification, ensuring transparent communication, and utilizing tamper-evident packaging or smart storage monitoring technologies [21, 27]. Bridging this perception gap is critical for transforming environmental concerns into actionable public support for sustainable pharmaceutical practices [21, 27].

Globally, the legality and feasibility of medication re-dispensing vary considerably across healthcare systems. Several countries have piloted or implemented medication reuse initiatives under strict regulatory and professional oversight to ensure product integrity and patient safety. For instance, the United Kingdom and the Netherlands have explored pharmacist-led reuse programs supported by tamper-evident packaging and digital monitoring systems to verify storage conditions and safety [13, 17, 18, 20]. In the United States, several states have enacted laws permitting the return and reuse of medications, primarily through safety-net or charitable programs that serve underserved populations [16]. Similarly, in Italy, a government-regulated program was piloted for 3 years, demonstrating the operational feasibility of medicine return and redistribution. Regionally, evidence from Jordan highlights strong public support for medication reuse, provided that safety and quality are ensured [21].

At the international level, the World Health Organization discourages the cross-border donation of unused medicines unless stringent quality and safety requirements are met [14]. These international and regional experiences collectively highlight both the potential advantages of reuse—such as cost savings, waste reduction, and improved access—and the operational and ethical challenges, including maintaining storage integrity, labeling accuracy, and public trust. In contrast, the Saudi Ministry of Health currently prohibits medication reuse on ethical grounds, and Saudi Arabia lacks a formal legal or regulatory framework to govern such practices. While lessons from other countries can inform local policy development, successful implementation in Saudi Arabia will require context-specific legislation, clear professional accountability, and public education to ensure safety, ethical compliance, and sustainability.

### Implications for practice and policy in Saudi Arabia

This study offers valuable insights that can guide the development of safe and acceptable medication reuse programs in Saudi Arabia. The following recommendations were proposed.

#### Create clear national guidelines for medication reuse

There is an urgent need for a formal regulatory framework to define which types of medications can be safely re-dispensed, how they should be assessed (e.g., expiry, packaging, and storage), and under what conditions this can occur. These guidelines should be developed by the Saudi Food and Drug Authority (SFDA) in collaboration with the Ministry of Health. They must align with international standards (e.g., European Medicines Agency (EMA), U.S. Food and Drug Administration (FDA), World Health Organization (WHO)) while being adapted to local legal, ethical, and cultural norms. Building such a framework will require a robust legal and regulatory infrastructure to safeguard patient safety and ensure ethical practice. Importantly, any reuse program must limit eligibility to medicines returned in their original, unopened manufacturer packaging with intact seals, complete labeling, and visible expiry dates. Excluding split-pack or bulk-supplied medicines is essential, as these often lack batch numbers or critical shelf-life information once opened, posing significant risks to safety and feasibility. By establishing clear criteria and strong governance, such a framework can guarantee both safety and credibility, while fostering public trust—an essential condition for the successful adoption of redispensing programs, particularly in contexts where medicines are scarce and in high demand.

#### Establish regional quality checking centers for returned medications

To ensure the safety, effectiveness, and integrity of returned medications before re-dispensing, regional quality checking units should be established across major regions, such as the Central, Western, and Eastern Provinces. These centers could operate in collaboration with licensed pharmacists and pharmaceutical companies and could be overseen by the Saudi Food and Drug Authority (SFDA). Their role includes quality testing of packaging, verifying expiry dates, and assessing storage conditions using innovative technologies. This step would help build public confidence in the reuse system and ensure compliance with the stringent safety standards.

#### Build public trust through transparent communication

Although many people in the study supported the idea of waste reduction, safety concerns made them hesitant to accept reused medications. Public education campaigns should be launched to explain how safety is ensured, including the role of pharmacists, the use of tamper-evident packaging, and verification steps. This can help shift attitudes from abstract support for sustainability to real willingness to use reused medications.

#### Empower pharmacists to lead the reuse process

Since pharmacist verification is the most important factor influencing public acceptance, pharmacists should be trained and positioned as the primary gatekeepers of any re-dispensing program. Their responsibilities include checking returned medicines, educating patients, and obtaining informed consent. Investing in their roles will also increase public confidence in the safety of reused medications.

#### Start with pilot programs and gradual expansion

To test the feasibility, small-scale pilot programs should be implemented in selected hospitals or community pharmacies. These pilots can focus on reusing noninvasive, low-risk medications, such as tablets and capsules. Evaluation of these pilots should include not only operational effectiveness but also public feedback, which can inform future policy and scaling efforts.

### Strengths and limitations

Strengths: This is among the first quantitative investigations in Saudi Arabia to assess public willingness to accept re-dispensed medications and identify the key factors influencing this acceptance. This research offers insights that can inform national policies aimed at reducing medication waste and enhancing sustainable pharmaceutical practices. Limitations: This study has some limitations. The reliance on convenience sampling and online distribution may have introduced selection bias, as individuals with Internet access, higher educational attainment, and greater interest in the topic were more likely to participate. As such, the findings may not fully reflect the perspectives of older adults, those with lower educational attainment, or individuals with limited digital literacy. Additionally, as the survey was primarily disseminated via social media platforms, individuals who are not active on these platforms may have been underrepresented, which could further limit the generalizability of the findings to less digitally engaged populations. Future studies could employ complementary recruitment methods (e.g., community-based or healthcare setting recruitment) to ensure the inclusion of less digitally active populations. Moreover, illiterate individuals were excluded due to the self-administered online format of the survey, which required the ability to read and respond independently. While necessary from a methodological standpoint, this exclusion may have restricted the representativeness of the sample. Furthermore, as the study was conducted in a single region, the findings may have limited generalizability beyond this region. The cross-sectional nature of this study captures public attitudes at a single point in time, limiting the ability to infer causality or track changes over time. Finally, since the data were self-reported, responses related to medication use, storage, and disposal practices may have been influenced by recall bias or social desirability.

## Conclusion

This study offers valuable insights into the public perception and acceptance of medication re-dispensing in Saudi Arabia. While overall support was cautious yet notable, particularly for oral solid dosage forms, willingness was highly dependent on safety assurances such as pharmacist verification, intact packaging, and informed consent. Key demographic factors, including age, employment status, regular prescription use, and attitudes toward medication waste, significantly influenced participants’ willingness to accept re-dispensed medications.

Interestingly, although many participants expressed concern about medication waste, this did not always translate into a higher willingness to use returned medicines, indicating a complex relationship between environmental awareness and perceptions of personal safety. Compared to findings from Western countries, Saudi respondents appeared to be more supportive of re-dispensing all types of medications, not just expensive ones, highlighting a potentially broader sense of social responsibility.

These findings suggest that the successful implementation of a medication reuse program in Saudi Arabia will require more than regulatory change; it will demand sustained public education, pharmacist engagement, and investment in safety and quality control measures. Pilot initiatives supported by strong policy frameworks and community trust-building efforts could serve as a foundation for scalable national programs to reduce waste and promote sustainable medicine use. At the same time, it is essential to recognize that medicines are not ordinary consumer products. Unlike items that can be reused without consequence, medicines have pharmacokinetic and pharmacodynamic properties that directly influence their safety, stability, and therapeutic effectiveness. Therefore, any future redispensing initiatives must be guided by strict professional and regulatory standards to ensure patient safety. Only medicines in their original, unopened manufacturer packaging with intact seals and visible expiry dates should be considered, and their reuse should occur under pharmacist supervision, supported by robust verification processes. By embedding such safeguards, redispensing programs can achieve their intended goals of reducing waste and promoting sustainability without compromising the quality and safety of patient care.

## Data Availability

The original contributions presented in the study are included in the article/supplementary material, further inquiries can be directed to the corresponding author.

## References

[1] Trueman P Lowson K Blighe A Mészáros Á Wright D Glanville JM Evaluation of the scale, causes and costs of waste medicines. York health economics consortium and school of pharmacy. London, United Kingdom: University of London (2010). Available online at: https://discovery.ucl.ac.uk/id/eprint/1350234/1/Evaluation_of_NHS_Medicines_Waste__web_publication_version.pdf (Accessed January 10, 2025).

[2] Paut Kusturica M Tomas A Sabo A . Disposal of unused drugs: knowledge and behavior among people around the world. Rev Environ Contam Toxicol (2017) 240:71–104. 10.1007/398_2016_3 27115675

[3] Shaaban H Alghamdi H Alhamed N Alziadi A Mostafa A . Environmental contamination by pharmaceutical waste: assessing patterns of disposing unwanted medications and investigating the factors influencing personal disposal choices. J Pharmacol Pharm Res (2018) 10(1):3.

[4] Garey KW Johle ML Behrman K Neuhauser MM . Economic consequences of unused medications in Houston, Texas. Ann Pharmacother (2004) 38(7-8):1165–8. 10.1345/aph.1D619 15138296

[5] Kadam A Patil S Patil S Tumkur A . Pharmaceutical waste management: an overview. Indian J Pharm Pract (2016) 9(1):2–8. 10.5530/ijopp.9.1.2

[6] Abou-Auda HS . An economic assessment of the extent of medication use and wastage among families in Saudi Arabia and arabian gulf countries. Clin Ther (2003) 25:1276–92. 10.1016/S0149-2918(03)80083-8 12809973

[7] Bekker CL Melis EJ Egberts ACG Bouvy M Gardarsdottir H van den Bemt B . Quantity and economic value of unused oral anti-cancer and biological disease-modifying anti-rheumatic drugs among outpatient pharmacy patients who discontinue therapy. Res Social Administrative Pharm (2019) 15:100–5. 10.1016/j.sapharm.2018.03.064 29610051

[8] Mackridge AJ Marriott JF . Returned medicines: waste or a wasted opportunity? J Public Health (2007) 29:258–62. 10.1093/pubmed/fdm037 17579236

[9] Toh MR Chew L . Turning waste medicines to cost savings: a pilot study on the feasibility of medication recycling as a solution to drug wastage. Palliat Med (2017) 31:35–41. 10.1177/0269216316639798 27430975

[10] Vogler S de Rooij R . Medication wasted—Contents and costs of medicines ending up in household garbage. Res Social Administrative Pharm (2018) 14:1140–6. 10.1016/j.sapharm.2018.02.002 29452743

[11] Bekker CL van den Bemt BJF Egberts ACG Bouvy ML Gardarsdottir H . Patient and medication factors associated with preventable medication waste and possibilities for redispensing. Int J Clin Pharm (2018) 40:704–11. 10.1007/s11096-018-0642-8 29721736 PMC5984955

[12] McRae D Allman M James D . The redistribution of medicines: could it become a reality? Int J Pharm Pract (2016) 24:411–8. 10.1111/ijpp.12275 27238215

[13] Bekker C van den Bemt B Egberts TC Bouvy M Gardarsdottir H . Willingness of patients to use unused medication returned to the pharmacy by another patient: a cross-sectional survey. BMJ Open (2019) 9(5):e024767. 10.1136/bmjopen-2018-024767 31092644 PMC6530303

[14] World Health Organization. Guidelines for drug donations. Geneva: World Health Organization (1999). Available online at: https://apps.who.int/iris/handle/10665/41901 (Accessed January 15, 2025).

[15] Bradshaw P Jarvis A . Reissuing returned medicines to developing countries. Pharm J (2004) 273:514–5.

[16] National Conference of State Legislatures. State prescription drug return, reuse, and recycling laws (2012). Available online at: http://www.ncsl.org/research/health/state-prescription-drug-return-reuseand-recycling.aspx (Accessed January 25, 2025).

[17] Gianino MM Cotugno V Scattaglia M Colasanto I Scaldaferri M Cattel F . Medicine recovery and reuse in a hospital setting: a lesson from Italy. Int J Pharm Pract (2022) 30(6):554–8. 10.1093/ijpp/riac056 35808979

[18] Bekker CL Gardarsdottir H Egberts TC Bouvy ML van den Bemt BJF . Redispensing of medicines unused by patients: a qualitative study among stakeholders. Int J Clin Pharm (2017) 39:196–204. 10.1007/s11096-017-0424-8 28070689

[19] Alhamad H Patel N Donyai P . How do people conceptualise the reuse of medicines? An interview study. Int J Pharm Pract (2018) 26:232–41. 10.1111/ijpp.12391 28795460 PMC5969265

[20] Hendrick A Baqir W Barrett S Prescribing Mrs Smith’s medication to Mr Jones: the views of patients and professionals on the reuse of returned medicines. Pharm Manag (2013) 29:25–26. 10.1136/bmjopen-2018-024767

[21] Alhamad H Jaber D Abu-Farha R Albahar F Edaily SM Donyai P . Factors influencing public willingness to reuse the unused stored medications in Jordan: a cross-sectional study. Healthcare (2022) 11(1):75. 10.3390/healthcare11010075 36611535 PMC9818750

[22] Lam Y McCrindle R Hui TKL Sherratt RS Donyai P . The effect of quality indicators on beliefs about medicines reuse: an experimental study. Pharmacy (Basel) (2021) 9(3):128. 10.3390/pharmacy9030128 34449720 PMC8396184

[23] Hui TKL Donyai P McCrindle R Sherratt RS . Enabling medicine reuse using a digital time temperature humidity sensor in an internet of pharmaceutical things concept. Sensors (Basel) (2020) 20(11):3080. 10.3390/s20113080 32485976 PMC7308820

[24] Hui TKL Mohammed B Donyai P McCrindle R Sherratt RS . Enhancing pharmaceutical packaging through a technology ecosystem to facilitate the reuse of medicines and reduce medicinal waste. Pharmacy (Basel) (2020) 8(2):58. 10.3390/pharmacy8020058 32244551 PMC7355753

[25] General Authority for Statistics. Population estimates in the midyear of 2021 (2021). Available online at: https://www.stats.gov.sa/sites/default/files/POP%20SEM2021E.pdf (Accessed January 30, 2025).

[26] McRae D Gould A Price-Davies R Tagoe J Evans A James DH . Public attitudes towards medicinal waste and medicines reuse in a ‘free prescription’ healthcare system. Pharmacy (Basel) (2021) 9(2):77. 10.3390/pharmacy9020077 33917990 PMC8167727

[27] Smale EM Egberts TCG Heerdink ER van den Bemt B Bekker C . Key factors underlying the willingness of patients with cancer to participate in medication redispensing. Res Social Administrative Pharm (2022) 18(12):3329–37. 10.1016/j.sapharm.2021.12.004 34973931

